# Sexual risk among Colombian adolescents: knowledge, attitudes, normative beliefs, perceived control, intention, and sexual behavior

**DOI:** 10.1186/s12889-018-6311-y

**Published:** 2018-12-17

**Authors:** Alexandra Morales, Pablo Vallejo-Medina, Daniella Abello-Luque, Alejandro Saavedra-Roa, Paola García-Roncallo, Mayra Gomez-Lugo, Eileen García-Montaño, Laurent Marchal-Bertrand, Janivys Niebles-Charris, Diana Pérez-Pedraza, José Pedro Espada

**Affiliations:** 1 Universidad Miguel Hernández, Elche, Spain; 2 Fundación Universitaria Konrad Lorenz, Bogotá, Colombia; 3 Corporación Universidad de la Costa, Barranquilla, Colombia

**Keywords:** Adolescents, Sexual behavior, Condom, Sexual risk, Colombia

## Abstract

**Background:**

Colombia has one of the highest rates of the human immunodeficiency virus (HIV) and pregnancies - both of which are influenced by lack of condom use -, among adolescent population in Latin America; however, the mechanisms underlying the inconsistent use of condoms in this population are poorly understood. This descriptive and cross-sectional study’s purpose was to examine sexual behavior and its precursors using the theory of planned behavior (TPB) and considering gender-based differences. Another objective was to study the mediating effect of intention in the relationship between behavior precursors and condom use based on the TPB.

**Methods:**

We recruited 1100 adolescents aged between 14 and 19 years old (*M* = 15.94, *SD* = 1.30, 54.4% female) from Bogotá and Barranquilla, two of the cities with highest adolescent birth rates among adolescents in Colombia. Sociodemographic variables, knowledge on HIV and other sexually transmitted infections (STIs), HIV/AIDS-related attitudes, including attitudes toward the use of condoms, normative beliefs, perceived behavioral control, behavioral intention, and sexual behavior were assessed using self-reports. All analyses were run using SPSS v25. The indirect effect of intention to explain the relationship between precursors and the use of condoms during sexual intercourse was estimated using the PROCESS v3 macro.

**Results:**

Descriptive analyses suggest a high risk of contracting sexually transmitted infections and unplanned pregnancies associated to inconsistent condom use, medium-low level of knowledge about sexual health, low normative beliefs regarding peers’ condom use, and a certain perceived difficulty for using condoms. Condoms are used 71% of the times they have sex, but only 22% of the participants use them consistently; girls use condoms more consistently than boys. Sexual risk characteristics differed significantly by gender. Mediation analyses indicated that condom use intention mediates the relationship between behavioral precursors and frequency of condom use, according to the TPB.

**Conclusions:**

Findings provide a better understanding of sexual risk and highlight important implications for the sexual and reproductive health of adolescents. There is a need of designing and implementing protocolized sexual health promotion programs in schools with the aim of reducing sexual risk behaviors in Colombian adolescents.

## Background

Unplanned pregnancies and sexually transmitted infections (STIs) among adolescents are public health problems all over the world, especially in low-income countries [1, 2]. One in five Colombian adolescents between 15 and 19 years of age is or has been pregnant; 64% of these pregnancies were not planned [3]. A total of 420,047 newborn children were registered in 2017, 20% of which (*n* = 86,239) were born from adolescents between 15 and 19 years of age [4]. In Colombia, the number of births per 1000 adolescents in the group of 15 to 19 years of age in 2015 (57.7) was lower than in countries of Central America such as Guatemala (84.0) and Nicaragua (92.8), but higher than others in South America such as Chile (52.1) and Peru (49.3) [5]. HIV/AIDS prevalence among Colombian youths is one of the highest in Latin America [2]. Rates for other STIs among young people between 15 and 24 years of age are also high, for instance, bacterial vaginosis (42%), human papillomavirus infection (28%), and infections by chlamydia (11.4%), gonorrhea (0.10%), and urethritis (6.2%) [6, 7].

Sexual and reproductive health decisions have important repercussions on adolescents’ physical and mental well-being (e.g. anxiety, depression, and alcohol/drug abuse). Worldwide data indicate that adolescent pregnancy is associated with medical problems during gestation and childbirth; it is also the second most important cause of death for girls between 15 and 19 years of age [1]. Adolescent mothers are more likely to be poor, mainly due to scarcer schooling and work opportunities than older mothers [3, 8, 9]. Finally, the adherence to antiretroviral treatment is lower in adolescents compared to adults, and it is associated with devastating consequences such as increased viral load, reduced defenses, and in the worst cases, death (see [10]).

Several social-cognitive models have attempted to identify variables predicting condom use, the most important protection method to avoid unplanned pregnancies and STIs after sexual abstinence. The theory of planned behavior (TPB) [11, 12] has been widely employed in meta-analytic and empirical studies on adolescent populations [13–15]. TPB states that the best predictor of behavior is intention to engage in the behavior; in turn, attitudes, normative beliefs, and perceived control predict intention. Therefore, adolescents who display a favorable attitude toward condoms, perceive that their peers use this protection method, and see themselves as capable of using condoms correctly will also likely have a firm intention to use condoms, and consequently, the behavior will be more likely to occur. Based on a recent study [15], the TPB seems to be the most suitable model for predicting condom use among young people.

Different studies in Colombia have found that adolescents often fail to use protection during sexual intercourse. Consistent use of condoms is estimated to be between 30 and 42%; girls are less likely than boys to use condoms [6, 16]. The prevalence of unprotected sex was revealed to be as high as 70% in a study with Colombian young people between 15 and 24 over the 12-month period before the study [16]. Becoming sexually active before 15, having low condom use intentions, and inadequate knowledge about STIs and protection methods are risk factors for lack of condom use identified for Colombian adolescent populations [6, 16, 17]. In a cohort study focused on students between 13 and 21 [6], investigators found that 60% of the students had become sexually active before 15 years of age, 58% failed to use condoms, and 39% had insufficient knowledge on sexual health.

The high prevalence of pregnancies and STIs among Colombian adolescents suggests that the current approach to sexual-affective education is inadequate [18, 19]. Therefore, more evidence of the mechanisms underlying sexual risk in this population is needed to tailor interventions to the specific characteristics of Colombian adolescents [16]. The scarce studies on sexual risk among adolescents in Colombia have focused on describing the sexual behaviors of college students; however, they rarely analyze the mechanisms behind risk behaviors using proven theoretical models (such as TPB). Further, small sample sizes from only one educational institution or geographical area prevent the generalization of results from these studies to other areas in the country.

The present study had two primary purposes: 1) to analyze factors associated with condom use (knowledge, attitudes, normative beliefs, perception of control, and intention) and sexual behavior in a large sample of Colombian adolescents using the TPB; and 2) to study role of intention concerning the use of condoms as a mediating agent between such precursor variables and the behavior itself. We hypothesize there will be a lack of consistency in using condoms and that girls use this method less than boys, as shown by the studies cited above. The present study also sought to confirm the relationships put forward by TPB using mediation analysis.

## Method

### Study design

This is a cross-sectional and descriptive study.

### Participants

The inclusion criteria to participate in the current study: (1) have both their informed written consent and their parents/legal tutors written informed consent completed, (2) being from 14 to 19 years old, and (3) attend to a school located in Bogota or Barranquilla areas. The exclusion criteria were: (1) to have a non-normal development and (2) not being able to read and write.

The sample was composed of 1100 adolescents between 14 and 19 years of age (*M* = 15.94; *SD* = 1.30). A slight majority (54.4%) were girls. All participants were Colombian high school students from the 9th to the 11th year who studied in different Colombian educational institutions. Six of these schools were located in Bogotá (*n* = 702; 63.8%) (Central area) and 7 in Barranquilla (*n* = 398; 36%) (Atlantic area). In Bogotá, four schools were private, one public and one mixed, while in Barranquilla 2 were private and five were public.

### Procedure

Snowball produce to contact centers was conducted. A total of 20 centers were approached initially. Incidental sampling was carried out in 13 Colombian educational institutions located in Bogotá and Barranquilla, two of the cities with highest birth rates among adolescents from 10 to 19 years of age [20]. Approval for the study was granted by the ethics committee of the Konrad Lorenz Foundation in Bogotá and by the Universidad de la Costa in Barranquilla. Educational institutions were informed about the purpose of the study and students from 14 to 19 years of age were invited to participate. All the participants signed the informed consent form; however, those from the ages 14 to 17 additionally presented parental authorization. Approximately, half of the informed consents were returned signed by parents. The questionnaire was administered in written form in groups of less than 35 students. Survey administrators were psychologists who had been trained to administer the questionnaire and visited the institutions during school hours. Participation was voluntary and anonymous, and participants were not given incentives of any kind.

### Measures

#### Sociodemographic variables

An ad hoc questionnaire was created to assess gender, age, nationality, city, educational institution, religion and attendance of religious services, family situation, and the number of participant’s children, and socioeconomic level (strata from 0 to 6). The strata is based on the fact that the significant housing-environment expresses a demonstrable socio-economic mode of life.

Based on the TPB [12], we assessed the following variables:

#### Knowledge about HIV and other STIs

Participants were administered the Colombian version of the Knowledge Scale on HIV and other STIs (ECI; [21]). It consists of 24 items divided by factor into five groups: general knowledge about HIV, knowledge about condoms, knowledge about forms of STI (including HIV) transmission, knowledge about STIs (including HIV) prevention, and knowledge about other STIs. An overall knowledge score is obtained from the sum of all item scores. In each item, the participant must respond to a statement by choosing one of three alternatives about the statement: true, false, or unknown. Higher scores represent more knowledge about HIV and other STIs. An item example is: *“Both the vaginal ring and the intrauterine device (copper “T”) are effective methods to prevent HIV/AIDS.”* The reliability of the scale for this sample was α = .74. In the current study, all subscales and the total score of the ECI were used.

#### Attitudes towards different aspects of HIV/AIDS

We used the Colombian version of the scale of attitudes toward aspects of HIV/AIDS (HIV-AS; [22]), which consists of 11 items. The response scale ranges from 1 *(Disagree completely)* to 4 *(Agree completely)*. The scale assesses four dimensions: attitudes toward the use of condoms when there are obstacles (e.g. the sexual partner does not want to use condom use or condoms are not available in the heat of the moment), attitudes toward the HIV test, attitudes toward the use of condoms in general, and attitudes toward people living with HIV/AIDS. High scores represent favorable (healthier) attitudes toward these aspects of HIV/AIDS. A sample item is: *“I would recommend a friend to take the HIV detection tests if they had been involved in risk practices (for instance, sex without using a condom).”* In the current study, the reliability of the subscales ranges between α = .64 and α = .70; overall scale reliability is α = .73. All subscales and the total score of the HIV-AS were used in the analyses.

#### Attitudes toward the use of condoms

An ad hoc seven-point Likert-type scale scoring five items was created to assesses participant perception of using condoms as a protection method in terms of very uncomfortable (1) – very comfortable (7); very harmful (1) – very health (7); very bad (1) – very good (7); very adverse (1) – very beneficial (7), and very unpleasant (1) – very pleasant (7). In the present study, the reliability of this instrument was excellent (α = .92). Individual items were used in the current study.

#### Normative beliefs

Four items assessed this concept: 1) “Do you think that people your age use condoms during sexual intercourse?” (*yes* or *no*); 2) “How often do you think your friends use condoms when they have sex?” (*Always, almost always, sometimes,* or *Never*); 3) “To what extent do you think that people who are important to you expect you to use a condom when you have sex?” (seven-point Likert scale from (1) *I am not expected to use a condom* to (7) *I am expected to use a condom*; 4) “To what extent are you willing to meet the expectations of people who are important to you?” (seven-point Likert scale from (1) *Not willing at all* a (7) *Completely willing*). Individual items were used in the current study.

#### Perception of control

This concept was assessed by three items using a seven-point Likert-type scale measuring the extent to which the participants felt they were capable of using a condom. 1) “How likely is it for you to use a condom?” (from 1- *Not likely at all* to 7- *Very likely*); 2) “How hard is it to use a condom?” (from 1- *Not hard at all* to 7- *Very hard*); and 3) “I am capable of using a condom” (from 1- *Not capable at all* to 7- *Very capable*). Internal consistency was not estimated for this measure because it included only three items evaluating aspects not related with one another.

#### Behavioral intention

Participants’ intention to engage in healthy sexual behaviors over the following 12 months was assessed. Examples of items are: *“*I will find condoms if I need them,” “I will use a condom if I have sex involving penetration,” (this individual item was used for the mediation analyses) and “I will tell the other person that we should use a condom before penetration” (in the current study; α = .73). The opposite, engaging in unhealthy sexual behaviors, was also assessed: “I will have sex after drinking too much alcohol,” and “I will have sex after taking other drugs (e.g., marihuana or acid)” (in the current study; α = .73)*.* The response scale is Likert-type with five points: 1 = *Definitely not*, 2 = *Probably not*, 3 = *Maybe*, 4 = *I probably will,* and 5 = *I definitely will.* Individual items were used in the current study.

#### Condom use

We assessed the percentage of condom use (“Please state the percentage of times when you use this protection method in your sexual relationships”), with a scale from 0 to 100%. Based on frequency of condom use, we calculated the variable of consistent use of condom (1 = consistent use: 100% of times; 0 = inconsistent use: less than 100%). The individual item was used in the current study.

Additionally, in order to study the sexual experience of the participants, different aspects of the adolescents’ relationship situation and sexual behavior and sexual orientation were assessed: 1) partner (“Do you currently have a partner?”) answers: *yes* or *no*; 2) age of sexual partner (years); 3) Sexual experience (“Have you ever had sexual interaction (oral, anal, or vaginal sex, or mutual masturbation?”; answers: *yes* or *no*; 4) number of sexual partners (“With how many people have you had sexual intercourse including penetration throughout your life?”); 5) having participated in any of the following five practices assessed individually: masturbation, mutual masturbation, oral sex, vaginal penetration, and anal penetration, responses *yes* or *no*; 6) age when the sexual practice was first tried (in case it has been tried) in years; 7) frequency of sexual relationships, with responses ranging from *I have not had sex* to *Every day or almost every day*; 8) use of condom in first intercourse (*I have not had sex*, *No*, *Yes,* or *I don’t remember*); and 9) sexual orientation (Kinsey’s scale); and 10) protection methods (Do you use any of the following protection methods in your penetrative sex? If yes, please indicate how many times you use this method of protection in your sexual relationships; options: condom, pills, no method, or other methods).

### Statistical analyses

All analyses were carried out using SPSS v25 statistical software. The study hypotheses were tested using the chi-square statistic for the analysis of categorical variables and Student’s *t* for quantitative variables. Sex differences in sociodemographic factors, condom use precursors, and sexual behavior were analyzed. Size of effect was calculated using odds ratio (*OR*) (for categorical variables) and Cohen’s *d* (for quantitative variables). Cohen’s *d* values ≤ .20 were considered very small, values between .21 and .49 we considered small, values between .50 and .79 were considered medium, and values ≥ .80 were considered large [23]. Reliability of the scales was calculated using Cronbach alpha. The significance level was set at α = .05 (95% confidence interval).

**Fig. 1:**
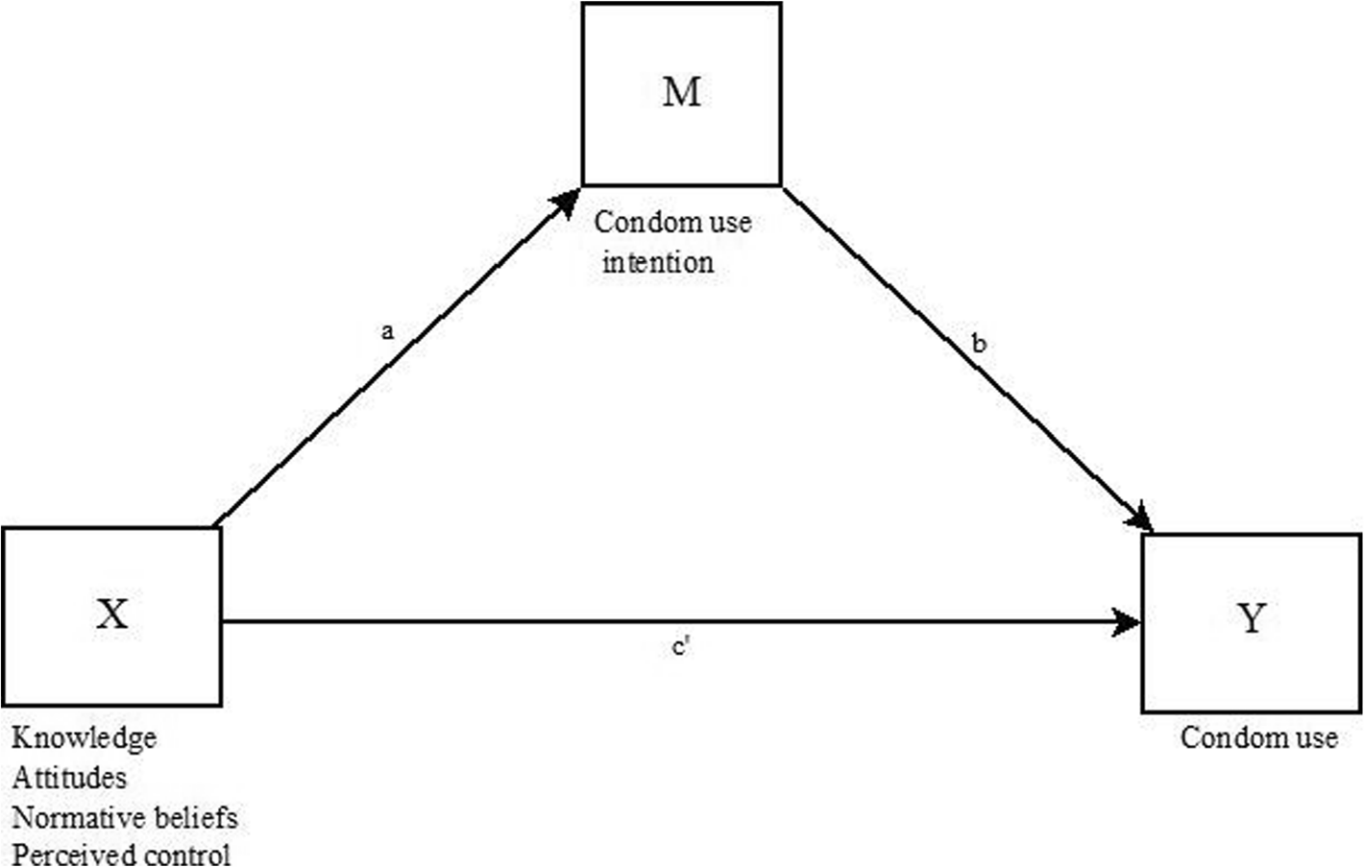
Path diagram of the effects of precursors (knowledge, attitudes, normative beliefs and perceived control) on condom use via condom use intention, after controlling for gender, age and educational center. X = Precursors. Y = Main outcome. M = Mediator. a, b, c = Regression coefficients. The 95% ACIs for indirect effects were obtained by bootstrapping with 5000 samples

Based on TPB, precursors (knowledge about HIV and other STIs, attitudes toward the use of condoms, normative beliefs, and perception of control) were included in mediation models as antecedent variables (X), whereas intention to use a condom was included as a mediating variable (M) (Fig. 1). Path α represented the effect of precursors on the intention to use condoms. Path β indicated the effect of the mediator on the result variable (Y = percentage of condom use). The indirect effect of condom use intention to explain the relationship between precursors and the use of condoms during sexual intercourse was estimated using the PROCESS v3 macro [24]. As described by recent studies, independent mediation models were calculated for each precursor [15, 25]. All models were adjusted for participants’ age and gender. The criterion to determine the effect of mediation was the lack of a zero in the confidence interval of the indirect effect.

## Results

### Participants

Table 1 summarizes the characteristics of the participants. Based on the Colombian socioeconomic level classification system, in which strata go from zero (lowest) to six (highest), the predominant strata were 2 (37.5%) and 3 (46.1%). A slight majority (61.6%) considered themselves as catholic. Half of the participants had married parents and 30.9% had divorced parents. Compared to females, males were slightly older (*p* = .05; *d* = .12), more likely to belong to a higher strata (*p* = .02; *d* = .19) and report to have at least one child (*p* = .01; *d* = .17). Compared to males, a greater proportion of females informed to be religious (mostly Catholic) and attend to religious events at least once a week (*p* = .01; *d* = .16).

**Table 1:** Sociodemographic variables of the participants and gender differences

	Females (*n* = 596)	Males (*n* = 504)	Total (*n* = 1100)	*p*	*d*
Age, *M* (*SD)*	15.87 (1.23)	16.03 (1.37)	15.94 (1.30)	.05	.12
14	84 (14.3)	61 (12.3)	145 (13.4)		
15	155 (26.4)	137 (27.6)	292 (27)		
16	170 (29)	131 (26.4)	302 (27.8)		
17	120 (20.4)	91 (18.3)	211 (19.5)		
18	45 (7.7)	45 (9.1)	90 (8.3)		
19	13 (2.2)	31 (6.3)	44 (4.1)		
Geographic area, *N* (%)					
Central area (Bogotá)	386 (64.9)	315 (62.5)	701 (63.8)	.40	–
Coast area (Barranquilla)	209 (35.1)	189 (37.5)	398 (36.2)		
Strata, *N* (%)					
0	2 (0.3)	0 (0)	2 (0.2)	.02	.19
1	74 (12.6)	46 (9.2)	121 (11.1)		
2	231 (39.2)	178 (35.5)	409 (37.5)		
3	260 (44.1)	242 (48.3)	502 (46.1)		
4	20 (3.4)	29 (5.8)	49 (4.5)		
5	2 (0.3)	6 (1.2)	8 (0.7)		
Religion, *N* (%)					
Christian	130 (21.9)	115 (22.9)	246 (22.4)	.01	.15
Catholic	387 (65.3)	288 (57.4)	676 (61.6)		
Buddhist	2 (0.3)	1 (0.1)	3 (0.3)		
Islamic	0 (0)	4 (0.8)	4 (0.4)		
None	58 (9.8)	75 (14.9)	133 (12.1)		
Other	16 (2.7)	19 (3.8)	35 (3.2)		
Attendance at religious events, *N* (%)					
Daily	19 (3.2)	18 (3.6)	37 (3.4)	.01	.16
At least once a week	160 (26.9)	107 (21.2)	267 (24.3)		
At least once every two weeks	51 (8.6)	41 (8.1)	92 (8.4)		
At least once every three weeks	39 (6.6)	21 (4.2)	60 (5.5)		
At least once a month	129 (21.7)	122 (24.2)	252 (22.9)		
At least once a year	128 (21.5)	104 (20.6)	233 (21.2)		
Never	68 (11.7)	91 (18.1)	159 (14.5)		
Family situation, *N* (%)					
Married parents	290 (48.9)	254 (50.8)	544 (49.8)	.32	–
Divorced parents	183 (30.9)	166 (33.2)	350 (31.9)		
Parents who live together	75 (12.6)	55 (11)	130 (11.9)		
Single father or mother	36 (6.1)	18 (3.6)	54 (4.9)		
Orphan of one parent or both	9 (1.5)	7 (1.4)	16 (1.5)		
Number of children, *M* (*SD*)	.01 (.10)	.05 (.30)	0.3 (0.22)	.01	.17

*M* Mean, *SD* Standard Deviation, *p* *p*-value, *d* Cohen’s *d*

### Knowledge about HIV and other STIs

The sampled population was shown to have a medium to low level of knowledge on HIV and other STIs (12.69 out of 24). Girls were more knowledgeable than boys, although the differences were significant only in the case of the general knowledge about HIV subscale and the overall score. Effect sizes were very small (Table 2).

**Table 2:** Condom use precursors and gender differences

Variables	Females	Males	Total	*p*	*d*
Knowledge, *M* (*SD*)					
HIV transmission (0–5)	2.56 (1.53)	2.71 (1.45)	2.64 (1.64)	.09	
Other STIs (0–6)	1.77 (1.78)	1.75 (1.80)	1.76 (1.79)	.84	
General HIV (0–8)	5.39 (1.45)	5.11 (1.51)	5.26 (1.48)	.002	.18
Condoms (0–2)	1.33 (.71)	1.37 (.72)	1.34 (0.71)	.36	
HIV prevention (0–3)	1.71 (1.11)	1.58 (1.10)	1.65 (1.11)	.06	
Total (0–24)	12.92 (4.23)	12.48 (4.73)	12.69 (4.35)	.05	.10
Attitudes towards aspects related to HIV, *M* (*SD*)					
Condom use (4–16)	13.54 (2.01)	13.34 (2.08)	13.45 (2.04)	.10	
Condom use when there are obstacles (3–12)	10 (1.76)	8.63 (2.09)	9.37 (2.03)	≤ .001	.70
HIV test (2–8)	7.12 (1.16)	6.75 (1.28)	6.95 (1.23)	≤ .001	.30
People living with HIV (2–8)	6.47 (1.43)	6.15 (1.52)	6.32 (1.48)	≤ .001	.21
Total (11–44)	37.15 (4.07)	34.90 (4.75)	36.12 (4.53)	≤ .001	.50
Condom as a method of protection is, *M* (*SD*)					
Very uncomfortable (1) - Very comfortable (7)	4.18 (2.24)	3.19 (1.98)	4.19 (2.12)	.95	–
Very harmful (1) - Very healthy (7)	4.98 (2.34)	5.15 (2.14)	5.06 (2.25)	.23	–
Very bad (1) - Very good (7)	4.94 (2.37)	4.98 (2.18)	4.96 (2.28)	.78	–
Very adverse (1) - Very beneficial (7)	5.11 (2.37)	5.13 (2.25)	5.12 (2.31)	.91	–
Not unpleasant (1) - Very pleasant (7)	3.83 (2.22)	3.82 (2.12)	3.83 (2.17)	.95	–
Normative beliefs on condom’s use					
Perception of peer’s condom use, *N* (%)	348 (59.1)	305 (62)	653 (60.4)	.33	–
Frequency use of peers’ condom use, *N* (%)					
Never	16 (2.7)	34 (6.9)	50 (4.6)	.005	.01
Sometimes	341 (58.4)	260 (52.6)	602 (55.9)		
Almost always	189 (32.4)	157 (31.9)	345 (32.1)		
Always	38 (6.5)	41 (8.3)	79 (7.3)		
People important to you expect you to use a condom (0–7), *M* (*SD*)	6.43 (1.13)	6.06 (1.29)	6.26 (1.22)	≤ .001	.30
Willing to meet the expectation of people who are important for you (0–7), *M* (*SD*)	5.56 (1.54)	5.82 (1.31)	5.68 (1.44)	.003	.20
Self-efficacy towards condom use, *M* (*SD*) (0–7)					
How likely is it that you use a condom correctly	2.90 (2)	3.03 (1.92)	2.96 (1.92)	.26	–
How hard is it to use a condom	4.80 (1.82)	5.02 (1.73)	4.90 (1.78)	.05	.12
I am capable of using a condom	5.88 (1.73)	5.77 (1.67)	5.83 (1.70)	.26	–
Behavioral intention, *M* (*SD*) (1–5)					
Look for a condom	3.72 (1.51)	3.94 (1.27)	3.82 (1.41)	.01	.15
Use a condom	4.42 (1.12)	4.38 (1)	4.40 (1.07)	.49	–
Condom use negotiation	4.40 (1.10)	4.16 (1.14)	4.29 (1.12)	≤ .001	.21
Have sex under the influence of alcohol	1.77 (.98)	2.19 (1.17)	1.96 (1.09)	≤ .001	.50
Have sex under the effect of drugs	1.44 (.88)	1.61 (1.08)	1.51 (0.98)	≤ .001	.17

*M* Mean, *SD* Standard Deviation, *p* *p*-value; *d* Cohen’s *d*

### Attitudes toward aspects of HIV/AIDS and toward the use of condoms

In general, favorable attitudes toward different aspects of HIV/AIDS were observed (36 out of 44). Girls’ attitudes were significantly more favorable than boys’ attitudes toward aspects of HIV/AIDS, using condoms when there are obstacles, the HIV test, and people living with HIV. Effect sizes of these differences ranged from small (*d* = .21) to medium (*d* = .70) (Table 2). In general, condoms as protection methods are considered moderately pleasant, healthy, good, and beneficial, although slightly uncomfortable to use during sex. There were no statistically significant gender-based differences concerning the specific attitude toward the characteristics of condoms.

### Normative beliefs on condom use

Six out of ten participants (60.4%) considered that their similar age peers use condoms during sexual intercourse. Most of them considered that their peers use condoms *sometimes* (55.9%) or *almost always* (32.1%). Only 7.3% of participants reported their belief that their peers *always* used condoms, whereas 4.6% considered that they *never* did (Table 2). In comparison with girls, a more substantial proportion of boys perceived that their peers *never* use condoms (2.7% vs. 6.9%) or always do it (6.5% vs. 8.3%).

In general, participants reported that people who are important to them expect them to use a condom during sexual intercourse (6.26 out of 7), and they reported being willing to meet the expectations of those people (5.68 out of 7). There were no gender-based differences between these two variables.

### Perceived control

The probability of using condoms correctly during sex involving intercourse was perceived as low (2.96 out of 7). Participants assessed the use of this protection method as moderately difficult (4.90 out of 7), although they perceived themselves as moderately capable of using it during vaginal, anal, and oral sex (if they wanted to) (5.83 out of 7) (Table 2).

### Behavioral intention

Participants reported a moderate to high intention of having a condom at hand (in case they need it), using the condom during sex, and negotiating its use with the sexual partner. Additionally, they reported little intention to engage in sex under the influence of alcohol or other drugs. As compared to girls, boys reported being more willing to obtain a condom, although they also reported being less willing to negotiate the use of the condom and more willing to have sex under the influence than girls. Effect sizes of these differences were small (*d* = .15) and moderate (*d* = .50) (Table 2).

### Sexual behavior and sexual orientation

Table 3 describes participants’ sexual behavior and differences by gender. The predominant sexual orientation was exclusively heterosexual (86.2%). One third of the sample (35.4%) reported having a couple sexual partners at the time of the survey, and 38.3% reported having had oral sex, vaginal penetration, or anal penetration at least once. The mean age of girls’ sexual partners was one year older than for boys’ sexual partners. Half of the participants (49.7%) reported having practiced masturbation, 37.2% vaginal sex, 29% oral sex, 26.6% mutual masturbation, and 8.2% anal penetration. Sexual activity initiation mean age was under 15 for all sexual practices, except anal penetration (*M* = 15.14; *SD* = 1.49). Thirty-three percent of participants who had practiced vaginal penetration began before 15, 32.9% in the case of oral sex, and 27.4% in the case of anal penetration. Condoms were the most widespread protection method (used 71.3% of the time for sex); however, only 22% of participants reported consistent condom use.

**Table 3:** Sexual behavior, sexual orientation, and gender differences

Variables	Females	Males	Total	*p*	*d / OR*
Have a sexual partner, *N* (%)	222 (37.4)	167 (33.1)	389 (35.4)	.13	–
Age of the sexual partner (0–45 years), *M* (*SD*)	17.31 (4.69)	16.27 (3.79)	16.87 (4.36)	.01	.22
Have had sex, *N* (%)	185 (31.2)	234 (46.9)	419 (38.3)	≤ .001	.51 [.40, .65]
Number of total sexual partners whom you have had penetration, *M* (*SD*)	.54 (1.10)	2.27 (11.50)	1.34 (7.89)	≤ .001	.21
Sexual orientation, *N* (%)					
Asexual	8 (1.4)	22 (4.5)	30 (2.8)	≤ .001	.11
Exclusively heterosexual	498 (84.3)	437 (88.5)	936 (86.2)		
Mainly heterosexual, with some sporadic homosexual contacts	44 (7.4)	9 (1.8)	53 (4.9)		
Mainly heterosexual, with several sporadic homosexual contacts	14 (2.4)	4 (0.8)	18 (1.7)		
Approximately the same homosexual and heterosexual contacts	15 (2.5)	11 (2.2)	26 (2.4)		
Mainly homosexual, with several sporadic heterosexual contacts	1 (0)	1 (0.2)	2 (0.2)		
Mainly homosexual, with some sporadic heterosexual contacts	5 (0.8)	1 (0.2)	6 (0.6)		
Exclusively homosexual	6 (1)	9 (1.8)	15 (1.4)		
Sexual practices, *N* (%)					
Masturbation	274 (46.6)	266 (53.5)	540 (49.7)	.02	.4 [.36, 62]
Mutual masturbation	121 (20.5)	169 (33.8)	290 (26.6)	≤ .001	1.9 [1.50, 2.50]
Oral sex	130 (22)	186 (37.39)	316 (29)	≤ .001	.47 [.36, .61]
Vaginal penetration	181 (30.6)	225 (45)	406 (37.2)	≤ .001	.53 [.42, .69]
Anal penetration	27 (4.6)	62 (12.5)	89 (8.2)	≤ .001	.33 [.21, .53]
Age of sexual initiation, *M* (*SD*)					
Masturbation	14.4 (1.5)	13.3 (1.9)	13.9 (1.8)	≤ .001	.65
Mutual masturbation	15.0 (1.4)	14.3 (1.9)	14.6 (1.7)	.002	.39
Oral sex	15.2 (1.4)	14.7 (1.5)	14.9 (1.5)	.004	.34
Vaginal penetration	15.1 (1.3)	14.5 (1.6)	14.8 (1.5)	≤ .001	.35
Anal penetration	15.7 (1.3)	14.8 (1.4)	15.1 (1.4)	.008	.64
Frequency of sexual relationships, *N* (%)					
I have not had sex	434 (73.6)	306 (62.2)	741 (68.4)	≤ .001	.14
1 time a month	55 (9.3)	88 (17.9)	143 (13.2)		
2 or 3 times a month	45 (7.6)	44 (8.9)	89 (8.2)		
1 or 2 times / week	33 (5.6)	30 (6.1)	63 (5.8)		
3 or more times/week	17 (2.9)	17 (3.5)	35 (3.1)		
All or almost every day	6 (1)	7 (1.4)	13 (1.2)		
Condom use at first sexual intercourse, *N* (%)					
I have not had sex	400 (67.3)	251 (50)	652 (59.4)	≤ .001	.30
I did not use it	47 (7.9)	71 (14.1)	118 (10.8)		
Yes	119 (20)	142 (28.3)	260 (23.7)		
I do not remember	28 (4.75)	38 (7.6)	54 (6.1)		
Methods of protection, *N* (%)					
Percentage of condom use, *M* (*SD*)	70.1 (31.43)	72.22 (25.86)	71.34 (28.40)	.41	
Consistent condom use, *N* (%)					
Yes	61 (26.4)	56 (18.5)	117 (22)	.03	1.57 [1.0, 2.38]
No	170 (73.6)	246 (81.5)	416 (78)		
Percentage of pill use, *M* (*SD*)	53.66 (38.71)	38.44 (33.51)	46.88 (37.18)	.004	.42
Other methods, *N* (%)					
No method	7 (31.8)	8 (40)	15 (35.7)	.43	–
Injection	7 (31.8)	6 (30)	13 (31)		
Implant	5 (22.7)	1 (5)	6 (14.3)		
Vaginal ring	1 (4.5)	0 (0)	1 (2.4)		
Postday	2 (9.2)	3 (15)	5 (11.8)		
Copper T	0 (0)	1 (5)	1 (2.4)		
Interrupted intercourse	0 (0)	1 (5)	1 (2.4)		

*M* Mean, *SD* Standard Deviation, *p* *p*-value, *d* Cohen’s *d*; *OR* = Odds Ratio

All the variables presented gender-based differences except for the percentage of participants who had a sexual partner (Table 3). Heterosexual relationships were predominant, although a significantly higher proportion of boys declared being exclusively heterosexual as compared with girls (88.5% vs. 84.3%). Boys were more likely to have had sexual intercourse than girls (46.9% vs. 31.2%). Boys were more likely to had masturbation, mutual masturbation, vaginal penetration, oral sex, and anal penetration than girls. Boys were more likely to initiate all practices earlier than girls, and had sex more frequently (*1 or 2 times/ week, 3 or more times/week, and All or almost every day*).

A more significant proportion of boys informed having used a condom during their first sexual intercourse in comparison with girls (28.3% vs. 20%). In general, boys reported using condoms more frequently than girls during sex (72.2% vs. 72.2% of the times they have sex), but girls reported more consistent use (26.4% vs. 18.5%).

### Condom use intention as a mediator between the precursors and condom use

Table 4 summarizes mediation analysis results. Path α shows a direct and significant relationship between precursors (attitude, normative beliefs, and perception of control) and intention to use condoms. Path β represents a direct and significant relationship between intention to use condoms and frequency of use in all models. A favorable attitude toward using condoms, having the perception that one’s peers use condoms in their sexual relationships, and thinking oneself as capable of using them correctly during sex ere associated with increased use, indirectly, through the intention of using condoms. Therefore, condom use intention was found to be a mediating variable between the studied precursors and the behavior in all models, except in the case of knowledge about HIV and other STIs.

**Table 4:** Mediating effect of the condom use intention in the relationship between the precursors (knowledge, attitudes, normative beliefs, and perceived control) and using a condom in sexual relations

	Effect of the precursor on condom use intention^d^			Effect of condom use intention in condom use			Indirect effect of condom use intention on the relationship between the precursors and condom use
Precursors of condom use intention	Path *α* ^a^ (SE)	95% CI	*p*	Path *β* ^b^ (SE)	95% CI	*p*	ACI^c^
Knowledge on HIV and STIs^e^	.01 (.01)	−.004, .03	.12	6.13 (1.20)	3.77, 8.49	**≤ .001**	.09 [−.03, .25]
Attitude towards condom use^f^	.13 (.02)	.08, .17	**≤ .001**	4.04 (1.19)	1.69, 6.40	**≤ .001**	**.53 [.16, 1.01]**
Normative beliefs on condom use^g^	.13 (.06)	.002, .25	**.04**	6.10 (1.22)	3.69, 8.51	**≤ .001**	**.79 [.04, 1.63]**
Perceived control on condom use^h^	.09 (.02)	.04, .14	**≤ .001**	5.12 (1.19)	2.79, 7.46	**≤ .001**	**.48 [.13, .98]**

ACI = Asymmetric confidence intervals according to the bootstrapping procedure with 5000 repetitions. The mediation analyzes were adjusted by gender, age and educational center

Statistically significant coefficients are indicated in bold

^a^Effect of each precursor in the condom use intention in sexual relationships

^b^Effect of condom use intention in the self-reported condom use (percentage of use)

^c^Effect of precursors on the behavior mediated by condom use intention (X - M- Y)

^d^Single item “I will use a condom if I have sex involving penetration” of the Colombian version of the HIV-AS

^e^Total score of the Colombian version of the Knowledge Scale on HIV and other STIs (ECI)

^f^Subscale attitudes toward the use of condoms of the Colombian version of the scale of attitudes toward aspects of HIV/AIDS

^g^Single item: “How often do you think your peers use condoms in their sexual relationships?” (Always, Almost always, Sometimes or Never)

^h^Single item: “I am able to use a condom” (from Not capable at all to Very capable)

## Discussion

The present study confirms that Colombian adolescents have a high risk of acquiring an STI or having an accidental pregnancy. Even though condoms are the most frequent protection method for sexual intercourse (used 71% of the time), only 22% of participants reported consistent use. The frequency of use in the present study was sensibly lower than the frequency measured in a sample of Spaniard students between 15 and 18 (87.1%); similar to the present research, the study made in Spain found a higher frequency of use among boys (93.5% vs. 80.8%) [26].

In our study, consistent use of condoms (22%) was less frequent than in other studies conducted in Colombia (30%) [16] and internationally (40–54%) [27, 28]. Navarro and Vargas [29] found that 82.1% of a sample of adolescents from Barranquilla, Colombia, used condoms occasionally. Valencia and Canaval [30] reported that 57.1% of Colombian youths had replaced condoms with other contraceptive methods, which suggests concern about becoming pregnant but not about acquiring an STI; a revealing piece of information in this study is that 30% of sexually active adolescents use contraceptive injections (administrated by a doctor or nurse in the health care system) despite that these are not an effective method against STIs.

Mediation analyses confirmed that the intention of using condoms was a “predictor” of the frequency with which this protection method is used during sexual intercourse. This finding is consistent with TPB [12] and with results of empirical studies that proved a model to predict the use of condoms among Spanish-speaking adolescents [15, 25]. The intention to enact healthy sexual behaviors (obtaining and using condoms and negotiating their use) was moderate to high in the present study.

The level of knowledge of HIV and other STIs was not associated with the intention of using condoms during sexual intercourse; these data are consistent with findings from the United States [31], Uganda [32] and Spain [25]. The level of knowledge on sexual health is a necessary precursor, although it is considered insufficient to predict the behavior by itself [15, 33]. In the present study, the level of knowledge of HIV and other STIs was medium to low; important gaps were observed regarding forms of transmission and general prevention. Other studies, focused on Colombian adolescents [17, 29] and Spaniard adolescents [26, 34], have reported similar results. This situation highlights the need to modify erroneous sexual beliefs and to provide reliable information to adolescents. For Latin American youths, the most important sources of information about sexuality are their parents (37.8%), followed by some other relative (17.1%), school (13.4%), and friends (11.4%) [35]. The knowledge gaps identified by our investigation can be explained by several reasons; for example, the lack of grasp on the matter of the principal information sources, the absence of conversation about sexual issues or even communication problems like misunderstandings.

Attitudes toward the use of condoms, perceived frequency of use among peers, and perceived control were found to be precursors of the intention to use condoms, and they were thus associated with the actual behavior of using condoms through intention. This statement is consistent with TPB [12] and international studies [15, 32]. The attitudes towards different aspects of HIV/AIDS (including condoms) were moderate to highly positive. Using the same evaluation instrument, Espada et al. [36] observed similar attitude patterns in Spaniard adolescents between ages 14 and 16.

On the other hand, 40% of Colombian adolescents think that their peers fail to use condoms during sexual intercourse, whereas only 7.3% believes that their peers use them consistently, which is very different from findings by Espada, Orgilés, Morales, Ballester, and Huedo-Medina [37], who reported that 43% of Spaniard students think that their peers use condoms consistently. According to the TPB [12], perceiving that their peers fail to protect themselves during sexual intercourse facilitates adolescents to decide not to protect themselves either. Nevertheless, Colombian adolescents believe that important people in their lives expect them to protect themselves during sexual intercourse, and most of them are willing to comply with these expectations.

Participants perceived themselves capable of using condoms when having sex (81.9%); however, 19.1% reported high difficulty to use them, and a considerable proportion (25%) considered their using condoms as unlikely. Given that a high perception of one’s capacity to control a behavior is associated with a higher probability of carrying it out [11], this aspect should be taken into account in the design of sexual health promotion interventions in the form of training on the correct use of condoms and elucidating the particular problem they have.

The gender-based differences observed in most of the analyzed variables suggest that sexual risk characterization varies by sex. Girls scored more favorably than boys in all areas except in their intention to find a condom, in which boys scored more favorably. Cultural beliefs associating the search of condoms with the desire to maintain sexual relationships may facilitate girls do not search for condoms (they prefer that boys provide them), so that they cannot be seen as they desire to have sex. According to this cultural belief, searching for condoms seems to be a protective behavior more expected in boys than girls, as stated by Valencia and Canaval [30].

As opposed to the study by Valencia and Canaval [30] (in which boys’ attitudes were more favorable than girls’), our research did not find sex differences in condom use attitudes. However, girls had a more favorable attitude toward the use of condoms when there are obstacles to use them compared to boys. Therefore, they could be expected to use the method despite their sexual partner’s resistance to using it or not having the condom at hand; this also explains their more consistent use of condoms for intercourse in comparison with boys.

More boys reported being sexually active and having initiated all sexual practices before 15 years of age. Even though sexual risk involves multiple factors, initiating sexual activity before 15 has been associated with higher risk of acquiring an STI and becoming prematurely pregnant [6, 38]. A higher proportion of boys inform to be sexually experienced and use more often condoms, but girls are more consistent in their use of condoms (18.5% vs. 26.4%). Consistent condom use rate was relatively low. Previous studies conducted with Colombian adolescents similar results; a higher proportion of males were sexually active compared to females [16] and they also used condom in a greater extent [30].

In the current study girls are more knowledgeable, display more favorable attitudes toward different aspects of HIV/AIDS, and find the use of condoms *easier* than boys do, and a higher percentage of girls believe that their peers are using condoms for sexual intercourse. According to the TPB, these precursors could explain why girls have stronger intentions to negotiate the use of condoms with their sexual partners, although their intentions to obtain them and use them are comparable to boys’. This unexpected result is possibly associated with cultural beliefs and values (e.g., *machismo* or *marianismo*) attributing boys the prerogative of procuring and producing the condom when engaging in sex, whereas girls are given a passive role (see [39]).

Thus, girls are better than boys at avoiding unplanned pregnancies and STIs. Girls’s couples mean age was higher than for boys’ couples; this could contribute in part to the observed sexual risk gender-based differences.

### Limitations

The results of the present study should be interpreted with caution and bear the limitations of this investigation in mind. Although the sample was broad and it included participants from schools in two different cities, it cannot be considered as representative of adolescents from Bogotá and Barranquilla, − nor – the Colombian population. Additionally, establishing causal relationships between the analyzed constructs was not possible due to the cross-sectional nature of the study. Even though cross-sectional data are suitable for testing theoretical models [40], it should be recognized that the relationship between the intention to use a condom and its actual use could have been overestimated as a result of using a retrospective measure instead of a prospective measure [15, 41]. Therefore, longitudinal studies would be in order to test the relationships between the constructs underlying sexual risk behaviors and analyze the evolution of such behaviors during adolescence. Finally, self-reports have a limited capacity to assess sexual behavior and are vulnerable to the effects of social desirability. Even though the use of biological measures (e.g., tests to detect HIV or other STIs) would provide objective data, the method is not cost-effective with samples in which the proportion of sexually active participants is low [42].

## Conclusions

One of the strengths of the present paper is its updating of sexual patterns among Colombian adolescents. These data provide a better insight into the potential reasons behind the high rates of STIs and unplanned pregnancies in this population. To the best of our knowledge, this is the first study to use the TPB for predicting the use of condoms for sexual intercourse in Colombian youths. Data indicate that Colombian adolescents are at a significant risk of becoming prematurely pregnant or acquiring an STI, mostly due to the inconsistent use of the method. This scenario confirms the shortfalls of preventive efforts in this area, which are not producing the expected results. The findings presented in this paper have significant implications for reproductive and sexual public health. These data can also be applied to the design of sexual health promotion interventions and to adapt programs of proven efficacy to Colombian populations. The present study also identified the necessity of putting protocolized sexual health promotion programs in place in educational institutions with the aim of reducing STIs and unplanned pregnancies among Colombian adolescents. Since the most important sources of information about sexuality for Latin American youths are their parents (37.8%), followed by some other relative (17.1%) [35], it is necessary to involve the family in sexual health promotion interventions.

## Abbreviations

*d*: Cohen’s *d*
HIV: Human immunodeficiency virus
*M*: Media
*SD*: Standard deviation
STIs: Sexually transmitted infections
TPB: Theory of planned behavior
